# Sequential Discrimination of Mixed Quantum States

**DOI:** 10.3390/e27030246

**Published:** 2025-02-27

**Authors:** Jin-Hua Zhang, Fu-Lin Zhang, Yan Gao, Wei Qin, Shao-Ming Fei

**Affiliations:** 1 Department of Physics, Xinzhou Normal University, Xinzhou 034000, China; zhjh216@cnu.edu.cn (J.-H.Z.); gaoyanfeifei@126.com (Y.G.); qinweiyuanyuan@163.com (W.Q.); 2 Department of Physics, School of Science, Tianjin University, Tianjin 300072, China; flzhang@tju.edu.cn; 3 School of Mathematical Sciences, Capital Normal University, Beijing 100048, China

**Keywords:** sequential discrimination, mixed state, mutual information

## Abstract

Classical mixtures of quantum states often give rise to decoherence and are generally considered detrimental to quantum processing. However, in the framework of sequential measurement, such mixtures can be beneficial for state discrimination. We investigate the sequential discrimination of mixed states and compare the results with those of pure states under the condition of equal fidelity. It is found that the successful probability of the mixed-state protocol is superior to the pure one under the equal-fidelity condition. It is shown that the difference between the sequential discrimination of pure and mixed states is more reliable under the equal-fidelity condition than under single-shot discrimination, and this difference increases with the mixability of the initial mixed states. For scenarios in which classical communication is allowed, the optimal successful probability of pure-state discriminations is larger than that for mixed states on the contrary. We also show that the classical mixture of basic vectors from quantum decoherence has a subtle impact on the communication channel induced by the coincidence of the maximal mutual information and optimal successful probability of sequential discrimination for pure states.

## 1. Introduction

Discrimination between quantum states has been a topic of much interest, having many applications in quantum information processing [1,2,3,4,5,6,7]. It is also a useful technique in quantum state manipulation and has deep connections to physical principles such as relativistic causality [8]. In the framework of quantum mechanics, as a fundamental consequence of the superposition principle, no measurement can discriminate between two nonorthogonal quantum states with certainty [9]. The challenge with quantum state discrimination is discriminating such states with the maximal success probability [10,11,12].

Based on unambiguous quantum state discrimination, Bergou et al. [13] developed a theory of nondestructive sequential quantum measurements, originating from the area of extracting information from a quantum system by multiple observers [14,15]. As a typical scenario in secure quantum communication strategies, the message sender Alice and the two subsequent observers, Bob and Charlie, may perform any premeasurement conspiracy, but no classical communication is allowed after Bob’s measurement. This is the protocol of sequential discrimination (SD) for quantum states. By avoiding classical communication, the quantum nature of the information is preserved, which is crucial for maintaining the integrity of quantum communication protocols such as the B92 quantum cryptography protocol [4].

Based on the joint measurement probability from both Bob and Charlie, the upper bound of the success probability is obtained for the initial states prepared with equal priors [13,16]. The results are then extended to the cases with prepared arbitrary prior pure states [17,18]. SD for more than three pure states [19] and more than two consecutive observers [20] has been also investigated with mixed initial states [21]. The upper bounds of the successful probabilities for the above results are both saturated when the part of information extracted by the first observer and the part left to the successor are equal.

Given the inevitable decoherence in quantum systems, mixed state discrimination becomes a crucial issue and has led to many novel outcomes [21,22]. In [22], the authors studied the single-shot discrimination of mixed states. An upper bound on the success probability was elegantly derived, which exhibited a linear correlation with the fidelity between the mixed states. A protocol for discriminating mixed states within the framework of sequential nondestructive measurements was also introduced in [21].

In order to determine the role played by quantum entanglement [23] and coherence [24] in state discriminations, researchers compared mixed state discrimination with the identification of a pair of pure states given by the basis vectors of the mixed states [23,24]. It was found that the pure–pure-state discrimination scheme is always superior to the mixed–mixed one for the protocol including only one observer [23,24].

However, these studies did not compare pure and mixed state discrimination in the framework of sequential measurement. This paper aims to fill this gap by showing that the classical mixture of state vectors may actually enhance the identification of quantum information, which contrasts the findings presented in [23,24].

Mutual information is used to quantify channel capacity or how much information can be transmitted between the involved parties [25]. The upper bounds of the mutual information between two successive observers, conditioned on the success results of the SD for the pure states, coincides with the optimal successful probability, making it a proper measure of information gain for the scheme that employs a sequential unambiguous discrimination strategy as a multiparty communication channel [18].

In this work, after we extend the calculation in [18] to the SD of mixed states, we consider the mutual information between the subsequent observers. We show that the optimal success probability of the SD for mixed states cannot be used as an ideal upper bound but an approximate estimate of the maximal mutual information. Moreover, the classical mixture of state vectors has a subtle influence on the mutual information in the framework of SD.

This paper is organized as follows. In Section 2 and Section 3, we present the result of SD for mixed states and compare the result with the one for pure states. In Section 4, we make a pure–mixed state discrimination comparison with respect to the scenarios including classical communications. In Section 5, we consider the mutual information in the SD of pure and mixed states separately and make a comparison. We summarize this paper in the last section.

## 2. Scheme for Sequential Mixed-State Discrimination

The concept of sequential discrimination (SD) for quantum states was initially proposed in [13]. The SD of pure states was conducted in the system-ancilla framework. The contributions of quantum correlations in this process were thoroughly explored in [16,17]. The SD of mixed states with the framework of optimal positive-operator-valued measurement (POVM) was presented in [21]. In this section, we outline the SD of mixed states in the system-ancilla framework.

An ensemble of two mixed states $\rho_{i}$ with a priori probability $P_{i}$ , i=1,2 , $P_{1}+P_{2}=1$ ( $P_{1}\leq 1/2$ ) is prepared and has a spectral decomposition,

(1) $$\rho_{i}=r_{i}|R_{i}\rangle \langle R_{i}|+{\tilde{r}}_{i}|{\tilde{R}}_{i}\rangle \langle {\tilde{R}}_{i}|,\;i=1,2,$$

with $r_{i},\;{\tilde{r}}_{i}\in \left[0,1\right]$ and $r_{i}+{\tilde{r}}_{i}=1$ . This mixed state is a statistical mixture of two vectors, $|R_{i}\rangle$ and $|{\tilde{R}}_{i}\rangle$ , with classical probabilities $r_{i}$ and ${\tilde{r}}_{i}$ , respectively. They fulfill the following relations:

(2) $$\langle R_{1}|R_{2}\rangle =s,\;\langle {\tilde{R}}_{1}|{\tilde{R}}_{2}\rangle =\tilde{s},\;\langle R_{i}|{\tilde{R}}_{i}\rangle =0,$$

where *s* and $\tilde{s}$ are real numbers such that $0<s,\tilde{s}<1$ .

The unambiguous discrimination of two general mixed states is difficult to handle and solve analytically [21]. In the following, we assume that the support spaces of the two states do not overlap, namely,

(3) $$\langle R_{1}|{\tilde{R}}_{2}\rangle =\langle {\tilde{R}}_{1}|R_{2}\rangle =0.$$

The relations in (3) indicate that the mixed state discrimination can be transformed into the discrimination of a pair of pure states, $|R_{1}\rangle$ , $|R_{2}\rangle$ and $|{\tilde{R}}_{1}\rangle$ , $|{\tilde{R}}_{2}\rangle$ , lying in their respective subspaces, which are orthogonal to each other [23].

The mixed states were discriminated via POVM measurements in [21,22]. According to the Neumark theorem, any realization of a specific POVM within a given space can always be achieved by expanding the original state space to a larger one and then carrying out the corresponding orthogonal measurements within this expanded space. In the study of quantum state discrimination, utilizing the auxiliary qubit framework may more effectively demonstrate the quantum properties involved [11,12,16,24].

After coupling to an auxiliary quantum system in state ${|0\rangle }_{b}$ , the mixed state discrimination procedure can be carried out via a joint unitary transformation $U_{b}$ on the primary state and the ancilla:

(4) $$U_{b}|R_{i}{\rangle |0\rangle }_{b}\;=\;|v_{i}{\rangle |\phi \rangle }_{b},\;\;U_{b}|{\tilde{R}}_{i}{\rangle |0\rangle }_{b}\;=\;|{\tilde{v}}_{i}\rangle |\tilde{\phi }\rangle_{b},$$

with

(5) $$\begin{array}{c} {|\phi \rangle }_{b}=\sqrt{q_{i}^{b}}{|0\rangle }_{b}+\sqrt{1-q_{i}^{b}}{|i\rangle }_{b},\;\\ |\tilde{\phi }\rangle_{b}=\sqrt{{\tilde{q}}_{i}^{b}}{|0\rangle }_{b}+\sqrt{1-{\tilde{q}}_{i}^{b}}{|\tilde{i}\rangle }_{b}, \end{array}$$

where $\{|0\rangle ,|i\rangle ,|\tilde{i}\rangle \}$ ( i=1,2 ) is the set of orthogonal bases of the ancilla and $|v_{i}\rangle$ and $|{\tilde{v}}_{i}\rangle$ are the post-measurement states of $|R_{i}\rangle$ and $|{\tilde{R}}_{i}\rangle$ , respectively.

**Figure 1:**
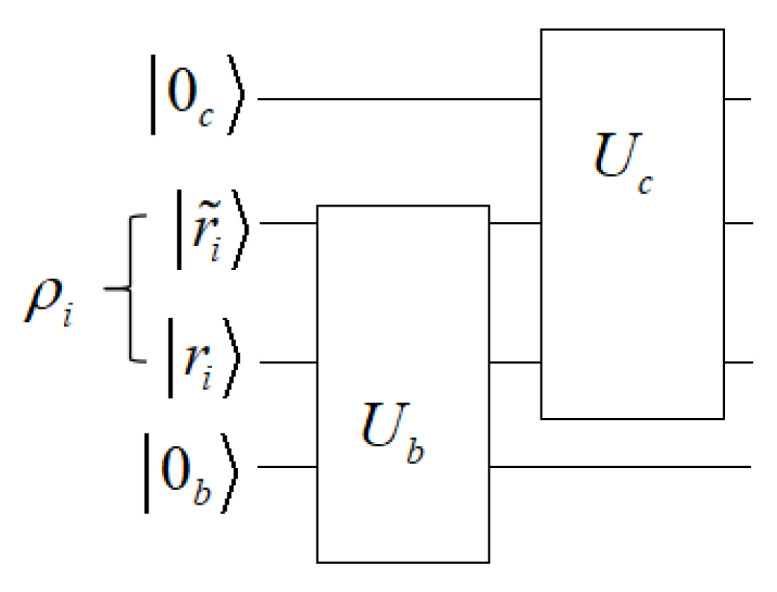
Protocol for SD. Alice has a quantum system *A* prepared in one of the two mixed states, $\rho_{1}$ and $\rho_{2}$ , with prior probabilities $P_{1}$ and $P_{2}$ , respectively. After Bob acquires the state, he performs a joint unitary operation between the principal and the auxiliary system *A* and *B*, respectively, followed by a von Neumann measurement on the ancilla. The states are successfully identified as $\rho_{1}$ ( $\rho_{2}$ ) if the measurement outcome is |1〉 or $|\tilde{1}\rangle$ ( |2〉 or $|\tilde{2}\rangle$ ). The discrimination fails if the measurement outcome is |0〉 . In this case, the post-measured state is sent to another observer, Charlie. Charlie performs a similar joint unitary operation $U_{c}$ on the principal and auxiliary systems and then optimally on a unambiguous state discrimination [13].

Denote the overlap $\langle v_{1}|v_{2}\rangle =t$ , $\langle {\tilde{v}}_{1}|{\tilde{v}}_{2}\rangle =\tilde{t}$ , with s≤t≤1 and $\tilde{s}\leq \tilde{t}\leq 1$ . Alice prepares a mixed state (1) and sends it to Bob, as shown in Figure 1. Generally, Bob’s measurement is nonoptimal in the sense that t≠1 and $\tilde{t}\neq 1$ . Namely, after Bob’s discrimination, there is some information left in the state. The post-measured state is then sent to another observer, Charlie.

Similar to Equation (4), Charlie makes a joint unitary operation $U_{c}$ on the principal system *A* and an auxiliary system *C* (in the initial state ${|0\rangle }_{c}$ ), with parameters $q_{i}^{b}$ and ${\tilde{q}}_{i}^{b}$ replaced with $q_{i}^{c}$ and ${\tilde{q}}_{i}^{c}$ , respectively. This is the so-called sequential discrimination (SD) [13,17,21] of quantum states without classical communications [13,16,17,21].

Since the inner product is conserved under the unitary operation, we have $\sqrt{q_{1}^{b}q_{2}^{b}}=s/t$ , $\sqrt{{\tilde{q}}_{1}^{b}{\tilde{q}}_{2}^{b}}=\tilde{s}/\tilde{t}$ , $\sqrt{q_{1}^{c}q_{2}^{c}}=t$ and $\sqrt{{\tilde{q}}_{1}^{c}{\tilde{q}}_{2}^{c}}=\tilde{t}$ . Then, the joint success probability of both Bob and Charlie identifying the states is acquired as

(6) $$\;\;P_{I}\;=\;\sum_{i=1}^{2}P_{i}\;\left[r_{i}\;\left(1\;\;-\;\;q_{i}^{b}\right)\;\left(1\;\;-\;\;q_{i}^{c}\right)\;\;+\;\;{\tilde{r}}_{i}\;\left(1\;\;-\;\;{\tilde{q}}_{i}^{b}\right)\;\left(1\;\;-\;\;{\tilde{q}}_{i}^{c}\;\right)\;\right].$$

After the scheme is equivalently implemented via POVM measurement, the result is the same as the one in Equation (6) (see Appendix A for further details). The optimization of the success probability is given by

(7) $$\begin{array}{c} \mathrm{maximize}:\;P_{I},\\ \mathrm{subject}\;\mathrm{to}:\;q_{1}^{c}q_{2}^{c}=q_{1}^{b}q_{2}^{b}=s^{2}, \end{array}$$

(8) $$\begin{array}{ccc} & & q_{1}^{b},q_{2}^{b},q_{1}^{c},q_{2}^{c}\in \left[s^{2},1\right],\;P_{1}\in \left(0,1/2\right]. \end{array}$$

The authors in [21] investigated the optimal success probability for the SD of mixed states and derived an equation of the POVM parameters that satisfied the optimal success probability. The explicit expression of the optimal success probability is obtained for the symmetric mixed state with equal prior ( $P_{1}=P_{2}=\frac{1}{2}$ , $r_{1}=r_{2}=r$ , ${\tilde{r}}_{1}={\tilde{r}}_{2}=\tilde{r}$ ). For arbitrary prior probabilities $P_{i}$ ( $\frac{P_{1}r_{1}}{P_{2}r_{2}}\leq 1$ ), we have the following analytical expressions of the optimal $P_{I}$ , occurring at $t=\sqrt{s}$ , $\tilde{t}=\sqrt{\tilde{s}}$ , $q_{1}^{b}=q_{1}^{c}$ , ${\tilde{q}}_{1}^{b}={\tilde{q}}_{1}^{c}$ :

$$\begin{array}{cc} (i):\; & P_{I}^{\max}=P_{2}r_{2}{\left(1-s\right)}^{2}+P_{2}{\tilde{r}}_{2}{\left(1-\tilde{s}\right)}^{2}, \end{array}$$

(9a) $$\begin{array}{cc} \; & \mathrm{when}\;s>s_{0},\;\tilde{s}>{\tilde{s}}_{0},q_{1}^{b}=q_{1}^{c}={\tilde{q}}_{1}^{b}={\tilde{q}}_{1}^{c}=1;\\ (\mathrm{ii}):\; & P_{I}^{\max}=P_{1}r_{1}{\left(1-q^{*}\right)}^{2}+P_{2}r_{2}{\left(1-\frac{s}{q^{*}}\right)}^{2}+P_{2}{\tilde{r}}_{2}{\left(1-\tilde{s}\right)}^{2}, \end{array}$$

(9b) $$\begin{array}{cc} \; & \mathrm{when}\;s\leq s_{0},\;\tilde{s}>{\tilde{s}}_{0},q_{1}^{b}=q_{1}^{c}=q^{*},{\tilde{q}}_{1}^{b}={\tilde{q}}_{1}^{c}=1;\\ (\mathrm{iii}):\; & P_{I}^{\max}=P_{2}r_{2}{\left(1-s\right)}^{2}+P_{1}{\tilde{r}}_{1}{\left(1-{\tilde{q}}^{*}\right)}^{2}+P_{2}{\tilde{r}}_{2}{\left(1-\frac{\tilde{s}}{{\tilde{q}}^{*}}\right)}^{2}, \end{array}$$

(9c) $$\begin{array}{cc} \; & \mathrm{when}\;s\leq s_{0},\;\tilde{s}>{\tilde{s}}_{0},q_{1}^{b}=q_{1}^{c}=1,{\tilde{q}}_{1}^{b}={\tilde{q}}_{1}^{c}={\tilde{q}}^{*};\\ (\mathrm{iv}):\; & P_{I}^{\max}=P_{1}r_{1}{\left(1-q^{*}\right)}^{2}+P_{2}r_{2}{\left(1-\frac{s}{q^{*}}\right)}^{2}+P_{1}{\tilde{r}}_{1}{\left(1-{\tilde{q}}^{*}\right)}^{2}+P_{2}{\tilde{r}}_{2}{\left(1-\frac{\tilde{s}}{{\tilde{q}}^{*}}\right)}^{2}, \end{array}$$

(9d) $$\begin{array}{cc} \; & \mathrm{when}\;s\leq s_{0},\;\tilde{s}\leq {\tilde{s}}_{0},q_{1}^{b}=q_{1}^{c}=q^{*},{\tilde{q}}_{1}^{b}={\tilde{q}}_{1}^{c}={\tilde{q}}^{*}. \end{array}$$

where $q^{*}$ , ${\tilde{q}}^{*}$ , $s_{0}$ and ${\tilde{s}}_{0}$ satisfy the following four equations, respectively:

(10) $$\begin{array}{cc} (i):\; & P_{1}r_{1}{\left(q^{*}\right)}^{4}-P_{1}r_{1}{\left(q^{*}\right)}^{3}+P_{2}r_{2}sq^{*}-P_{2}s^{2}=0;\\ \;(\mathrm{ii}):\; & P_{1}{\tilde{r}}_{1}{\left({\tilde{q}}^{*}\right)}^{4}-P_{1}{\tilde{r}}_{1}{\left({\tilde{q}}^{*}\right)}^{3}+P_{2}{\tilde{r}}_{2}s{\tilde{q}}^{*}-P_{2}s^{2}=0;\\ \;(\mathrm{iii}):\; & P_{1}r_{1}{\left(1-q^{*}\right)}^{2}+P_{2}r_{2}{\left(1-\frac{s_{0}}{q^{*}}\right)}^{2}=P_{2}r_{2}{\left(1-s_{0}\right)}^{2};\\ \;(\mathrm{iv}):\; & P_{1}{\tilde{r}}_{1}{\left(1-{\tilde{q}}^{*}\right)}^{2}+P_{2}{\tilde{r}}_{2}{\left(1-\frac{{\tilde{s}}_{0}}{{\tilde{q}}^{*}}\right)}^{2}=P_{2}{\tilde{r}}_{2}{\left(1-{\tilde{s}}_{0}\right)}^{2}. \end{array}$$

For $s\leq s_{0}$ and $\tilde{s}\leq {\tilde{s}}_{0}$ , we have $q_{1}^{b}=q_{1}^{c}=q^{*}$ ( ${\tilde{q}}_{1}^{b}={\tilde{q}}_{1}^{c}={\tilde{q}}^{*}$ ), and $q_{1}^{b}=q_{1}^{c}=1$ ( ${\tilde{q}}_{1}^{b}={\tilde{q}}_{1}^{c}=1$ ) for $s>s_{0}$ ( $\tilde{s}>{\tilde{s}}_{0}$ ), corresponding to the optimal SD of mixed states. In the former case, both mixed states are identified by Bob and Charlie. For the latter case, the success probability of both $|r_{1}\rangle \langle r_{1}|$ and $|{\tilde{r}}_{1}\rangle \langle {\tilde{r}}_{1}|$ equals zero [17,21]. If only one of $|r_{1}\rangle \langle r_{1}|$ and $|{\tilde{r}}_{1}\rangle \langle {\tilde{r}}_{1}|$ is omitted and the other one is identified, the SD of $\rho_{1}$ is partially successful (e.g., the case for $s\leq s_{0}$ and $\tilde{s}>{\tilde{s}}_{0}$ , the optimal success probability is acquired at $q_{1}^{b}=q_{1}^{c}=\sqrt{s}$ and ${\tilde{q}}_{1}^{b}={\tilde{q}}_{1}^{c}=1$ ). For the symmetric mixed state with equal prior, it can be easily acquired that $s_{0}={\tilde{s}}_{0}=3-2\sqrt{2}$ , $q^{*}=\sqrt{s}$ and ${\tilde{q}}^{*}=\sqrt{\tilde{s}}$ . Then, the optimal success probability shown in Table 1 is the same as the results in Table I in Ref. [21].

## 3. Comparison of Sequential Mixed State with Pure State Discriminations

To show the essential difference between the classical mixture and the quantum superposition in the framework of SD, rather than the protocol including only one observer given in [23], we consider the SD of the following states:

(11) $$|\Psi_{i}\rangle \;=\;\sqrt{r_{i}}|R_{i}\rangle \;+\;\exp\left(i\phi_{i}\right)\sqrt{{\tilde{r}}_{i}}|{\tilde{R}}_{i}\rangle ,\;i=1,2,$$

which are coherently superposed by the basic vectors $|R_{i}\rangle$ and $|{\tilde{R}}_{i}\rangle$ with probability amplitudes *r*, $\tilde{r}$ , and the phase factor $\phi_{i}$ , occurring with prior probability $P_{i}$ . The inner product of $|\Psi_{1}\rangle$ and $|\Psi_{2}\rangle$ is

$$s^{*}=\langle \Psi_{1}|\Psi_{2}\rangle =\sqrt{r_{1}r_{2}}s+\exp\left[i\left(\phi_{2}-\phi_{1}\right)\right]\sqrt{{\tilde{r}}_{1}{\tilde{r}}_{2}}\tilde{s}.$$

Bob and Charlie need to discriminate $|\Psi_{1}\rangle$ and $|\Psi_{2}\rangle$ sequentially. Bob’s unitary operation is given by

(12) $$U_{b0}|\Psi_{i}{\rangle |0\rangle }_{b}=|\Phi_{i}\rangle |\alpha_{i}^{0}\rangle_{b},$$

where $|\alpha_{i}^{0}\rangle_{b}=\sqrt{q_{Bi}^{0}}{|0\rangle }_{b}+\sqrt{1-q_{Bi}^{0}}{|i\rangle }_{b}$ , i=1,2 , and _b_ ${\langle \Phi_{1}|\Phi_{2}\rangle }_{b}=t^{*}$ . After a von Neumann measurement, the state is sent to Charlie. Charlie’s operation is of the same form as Bob’s, with parameters $q_{Bi}^{0}$ replaced with $q_{Ci}^{0}$ .

Then, the optimal success probability of SD for $|\Psi_{1}\rangle$ and $|\Psi_{2}\rangle$ is obtained by maximizing

(13) $$\begin{array}{ccc} P_{\mathrm{II}} & = & \sum_{i=1}^{2}P_{i}\left(1-q_{Bi}^{0}\right)\left(1-q_{Ci}^{0}\right) \end{array}$$

subject to

(14) $$\begin{array}{ccc} P_{1} & \in & \left(0,1/2\right],\;q_{B1}^{0}q_{B2}^{0}=|s^{*}{|}^{2}/{\left|t^{*}\right|}^{2},\\ q_{C1}^{0}q_{C2}^{0} & = & |t^{*}{|}^{2},\;q_{B1}^{0},q_{B2}^{0}\in \left[\right|s^{*}{|}^{2}/|t^{*}{|}^{2},1],\\ q_{C1}^{0},q_{C1}^{0} & \in & [|t^{*}{|}^{2},1]. \end{array}$$

Then, for arbitrary prior probability $P_{1}\leq \frac{1}{2}$ , the optimal joint success probability, which occurs at $|t^{*}|=\sqrt{|s^{*}|}$ and $q_{B1}^{0}=q_{C1}^{0}$ , is [17]

(15a) $$\begin{array}{ccc} (i):\; & P_{\mathrm{II}}^{\max}=P_{1}{\left(1-q_{0}^{*}\right)}^{2}+P_{2}{\left(1-\frac{|s^{*}|}{q_{0}^{*}}\right)}^{2} & \mathrm{for}\;0<|s^{*}|\leq s_{0}^{*}, \end{array}$$

(15b) $$\begin{array}{ccc} (\mathrm{ii}):\; & P_{\mathrm{II}}^{\max}=P_{2}\left(1-\right|s^{*}{\left|\right)}^{2} & \mathrm{for}\;s_{0}^{*}<\left|s^{*}\right|<1, \end{array}$$

where $q_{0}^{*}$ satisfies $P_{1}q_{0}^{*4}-P_{1}q_{0}^{*3}+P_{2}sq_{0}^{*}-P_{2}s^{2}=0$ , and the critical value $s_{0}^{*}$ satisfies $P_{1}{\left(1-q_{0}^{*}\right)}^{2}+P_{2}{\left(1-\frac{|s_{0}^{*}|}{q_{0}^{*}}\right)}^{2}=P_{2}\left(1-\right|s_{0}^{*}{\left|\right)}^{2}.$ For case (i), the optimal success probability is achieved when $q_{B1}^{0}=q_{C1}^{0}=q_{0}^{*}$ , whereas for case (ii), the optimal success is attained at $q_{B1}^{0}=q_{C1}^{0}=1$ , where Bob and Charlie conspire to ignore state $|\Psi_{1}\rangle$ .

For the SD of the pure states $|\Psi_{1}\rangle$ and $|\Psi_{2}\rangle$ , which are prepared with equal prior probabilities ( $P_{1}=P_{2}=\frac{1}{2}$ ), the result presented in Equation (15) simplifies to [16,17]

(16a) $$\begin{array}{ccc} (i):\; & P_{\mathrm{II}}^{\max}={\left(1-\sqrt{|s^{*}|}\right)}^{2} & \mathrm{for}\;|s^{*}|\leq 3-2\sqrt{2}, \end{array}$$

(16b) $$\begin{array}{ccc} (\mathrm{ii}):\; & P_{\mathrm{II}}^{\max}=1/2(1-|s^{*}{\left|\right)}^{2} & \mathrm{for}\;|s^{*}|>3-2\sqrt{2}, \end{array}$$

attained at $q_{Bi}^{0}=q_{Ci}^{0}=\sqrt{|s^{*}|}$ ( $q_{B1}^{0}=q_{C1}^{0}=1$ ) for $0<|s^{*}|\leq 3-2\sqrt{2}$ ( $3-2\sqrt{2}<\left|s^{*}\right|<1$ ). To compare in detail the optimal success probabilities of SD between the pure and symmetric mixed states ( $r_{1}=r_{2}=r$ , prepared with equal prior), we first introduce the following lemma.

**Lemma** **1.** ∀ x∈(0,1) *, we have ${\left(1-\sqrt{x}\right)}^{2}\geq 1/2{\left(1-x\right)}^{2}$ if $0<x\leq 3-2\sqrt{2}$ , and ${\left(1-\sqrt{x}\right)}^{2}<1/2{\left(1-x\right)}^{2}$ if $3-2\sqrt{2}<x<1$ .*

**Proof.**  Set

$$F\left(x\right)={\left(1-\sqrt{x}\right)}^{2}-\frac{1}{2}{\left(1-x\right)}^{2}.$$

We have

(17) $$F\left(x\right)={\left(1-\sqrt{x}\right)}^{2}\left[1+\frac{\sqrt{2}}{2}\left(1+\sqrt{x}\right)\right]f\left(x\right)$$

with $f\left(x\right)=1-\frac{\sqrt{2}}{2}\left(1+\sqrt{x}\right)$ . It can be easily found that f(x)≥0 ( f(x)<0 ) for $0<x\leq 3-2\sqrt{2}$ ( $3-2\sqrt{2}<x<1$ ). In addition, according to Equation (17), it is easily found that F(x) and f(x) have the same positive and negative signs. □

The fidelity $F(|\Psi_{1}\rangle ,|\Psi_{2}\rangle )$ between the coherent superposed states $|\Psi_{1}\rangle$ and $|\Psi_{2}\rangle$ and the fidelity between the mixed states $\rho_{1}$ and $\rho_{2}$ have the following relation:

(18) $$\begin{array}{ccc} F(|\Psi_{1}\rangle ,|\Psi_{2}\rangle ) & = & |s^{*}|=|rs+\exp\left[i\left(\phi_{2}-\phi_{1}\right)\right]\tilde{r}\tilde{s}|\\ & \leq & rs+\tilde{r}\tilde{s}=F\left(\rho_{1},\rho_{2}\right). \end{array}$$

When $\phi_{2}=\phi_{1}+2k\pi$ for some integer *k*, since $0<s^{*}<1$ , we have

(19) $$F(|\Psi_{1}\rangle ,|\Psi_{2}\rangle )=F\left(\rho_{1},\rho_{2}\right).$$

Then, under this equal-fidelity condition, we have the following theorem.

**Theorem** **1.** *The optimal success probability $P_{\mathrm{II}}^{\max}$ of the SD for $|\Psi_{1}\rangle$ and $|\Psi_{2}\rangle$ given in (11) is inferior to $P_{I}^{\max}$ of the SD for $\rho_{1}$ and $\rho_{2}$ given in (1).*

**Figure 2:**
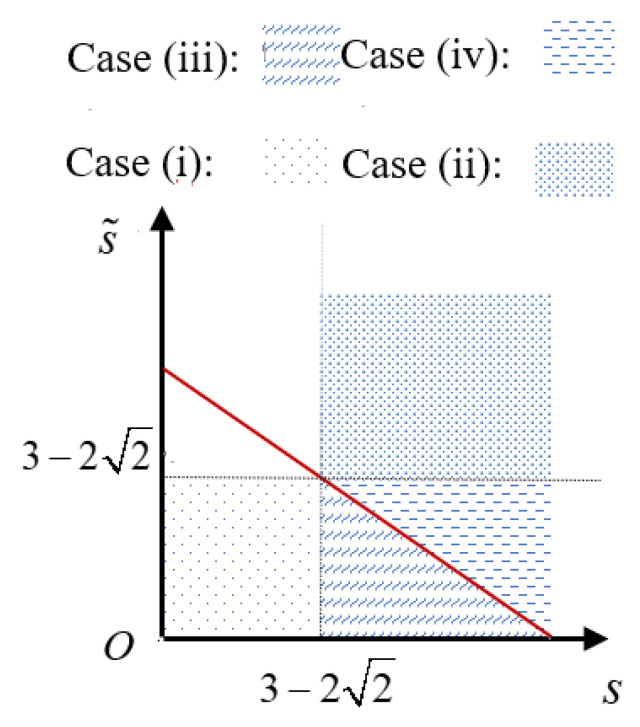
Four regions corresponding to cases (i), (ii), (iii), and (iv) for different values of *s*, $\tilde{s}$ , and $s^{*}$ . The red solid line is given by $s^{*}=rs+\tilde{r}\tilde{s}=3-2\sqrt{2}$ with fixed *r* and $\tilde{r}$ .

**Proof** **of** **Theorem** **1.** The corresponding SD results for mixed states are shown in Table 1; the four cases with respect to different value ranges of *s*, $\tilde{s}$ and $s^{*}$ are illustrated in Figure 2.

Case (i) ( $s\leq 3-2\sqrt{2}$ , $\tilde{s}\leq 3-2\sqrt{2}$ ). We easily obtain that $s^{*}=rs+\tilde{r}\tilde{s}\leq 3-2\sqrt{2}$ . The difference between the optimized successful probabilities of SD for pure and mixed states is given by

(20) $$\begin{array}{ccc} \Delta P & = & P_{I}^{\max}-P_{\mathrm{II}}^{\max}\\ & = & r{\left(1-\sqrt{s}\right)}^{2}+\tilde{r}{\left(1-\sqrt{\tilde{s}}\right)}^{2}-{\left(1-\sqrt{s^{*}}\right)}^{2}\\ & = & \frac{r\tilde{r}{\left(\sqrt{s}-\sqrt{\tilde{s}}\right)}^{2}}{\sqrt{rs+\tilde{r}\tilde{s}}\left(r\sqrt{s}+\tilde{r}\sqrt{\tilde{s}}\right)}\geq 0. \end{array}$$

Case (ii) ( $s>3-2\sqrt{2}$ , $\tilde{s}>3-2\sqrt{2}$ ). We have $s^{*}>3-2\sqrt{2}$ . According to Equation (16b), we have

(21) $$\begin{array}{ccc} \Delta P & = & P_{I}^{\max}-P_{\mathrm{II}}^{\max}\\ & = & \frac{1}{2}r{\left(1-s\right)}^{2}+\frac{1}{2}\tilde{r}{\left(1-\tilde{s}\right)}^{2}-\frac{1}{2}{\left(1-s^{*}\right)}^{2}\\ & = & r\tilde{r}{\left(s-\tilde{s}\right)}^{2}\geq 0. \end{array}$$

Case (iii) ( $s>3-2\sqrt{2}$ , $\tilde{s}\leq 3-2\sqrt{2}$ , $s^{*}\leq 3-2\sqrt{2}$ ). Since $s>3-2\sqrt{2}$ and $s^{*}\leq 3-2\sqrt{2}$ , from *Lemma 1*, one obtains

$$\frac{1}{2}r{\left(1-s\right)}^{2}>r{\left(1-\sqrt{s}\right)}^{2}.$$

Then,

(22) $$\begin{array}{ccc} \Delta P & = & P_{I}^{\max}-P_{\mathrm{II}}^{\max}\\ & = & \frac{1}{2}r{\left(1-s\right)}^{2}+\tilde{r}{\left(1-\sqrt{\tilde{s}}\right)}^{2}-{\left(1-\sqrt{s^{*}}\right)}^{2}\\ & > & r{\left(1-\sqrt{s}\right)}^{2}+\tilde{r}{\left(1-\sqrt{\tilde{s}}\right)}^{2}-{\left(1-\sqrt{s^{*}}\right)}^{2}\\ & = & \frac{r\tilde{r}{\left(\sqrt{s}-\sqrt{\tilde{s}}\right)}^{2}}{\sqrt{rs+\tilde{r}\tilde{s}}\left(r\sqrt{s}+\tilde{r}\sqrt{\tilde{s}}\right)}\geq 0. \end{array}$$

Case (iv) ( $s>3-2\sqrt{2}$ , $\tilde{s}\leq 3-2\sqrt{2}$ , $s^{*}>3-2\sqrt{2}$ ). According to $\tilde{s}\leq 3-2\sqrt{2}$ and *Lemma 1*, one has

$$\frac{1}{2}\tilde{r}{\left(1-\tilde{s}\right)}^{2}\leq \tilde{r}{\left(1-\sqrt{\tilde{s}}\right)}^{2}.$$

Therefore,

(23) $$\begin{array}{ccc} \Delta P & = & P_{I}^{\max}-P_{\mathrm{II}}^{\max}\\ & = & \frac{1}{2}r{\left(1-s\right)}^{2}+\tilde{r}{\left(1-\sqrt{\tilde{s}}\right)}^{2}-\frac{1}{2}{\left(1-s^{*}\right)}^{2}\\ & \geq & \frac{1}{2}r{\left(1-s\right)}^{2}+\frac{1}{2}\tilde{r}{\left(1-\tilde{s}\right)}^{2}-\frac{1}{2}{\left(1-s^{*}\right)}^{2}\\ & = & \frac{1}{2}r\tilde{r}{\left(s-\tilde{s}\right)}^{2}\geq 0. \end{array}$$

Owing to the symmetry in swapping *s* and $\tilde{s}$ , another scenario of a one-state partially identified case, where $s\leq 3-2\sqrt{2}$ , ${\tilde{s}}_{0}>3-2\sqrt{2}$ , results in the same conclusions for the optimal SD of mixed states to those in cases (iii) and (iv). □

Obviously, this conclusion of Theorem 1 is quite different from the results in Refs. [23,24]. That is, interestingly, in the framework of SD, the classical mixture of state vectors that brings about decoherence intensifies SD, on the contrary. In the discrimination of pure states $|\Psi_{1}\rangle$ and $|\Psi_{2}\rangle$ , the overlap of $|\Psi_{1}\rangle$ and $|\Psi_{2}\rangle$ quantifies the information encoded in the states (negative correlation) [16]. Corresponding to the optimal solution of the SD for $|\Psi_{1}\rangle$ and $|\Psi_{2}\rangle$ , after the first measurement, we set $F_{1}=\sqrt{s^{*}}$ , which denotes the residual information. It equals the one extracted by the first observer. Since the success probability of the SD for mixed states is equivalent to a weighted average of the one for two pairs of pure states $|r_{1}\rangle$ , $|r_{2}\rangle$ , $|{\tilde{r}}_{1}\rangle$ , and $|{\tilde{r}}_{2}\rangle$ [23], the information left in the quantum system after the first measurement in the procedure for the optimal sequential discrimination of the mixed states $\rho_{1}$ and $\rho_{2}$ can be expressed as $F_{2}=r\sqrt{s}+\tilde{r}\sqrt{\tilde{s}}.$ Hence,

(24) $$F_{1}^{2}-F_{2}^{2}=r\left(1-r\right){\left(\sqrt{s}-\sqrt{\tilde{s}}\right)}^{2}\geq 0.$$

Thus, there is more information left after the first measurement for the optimal SD of mixed states than for pure states. The extracted and residual information is no longer equivalent, which results in the conclusion of [*Theorem 1*]. As $s=\tilde{s}$ , the relation (24) is a real equation and we have that $P_{\mathrm{II}}^{\max}=P_{I}^{\max}$ . Thus, one concludes that the superiority of SD for mixed states also depends on the symmetry of information distribution encoded in the mixed state.

**Figure 3:**
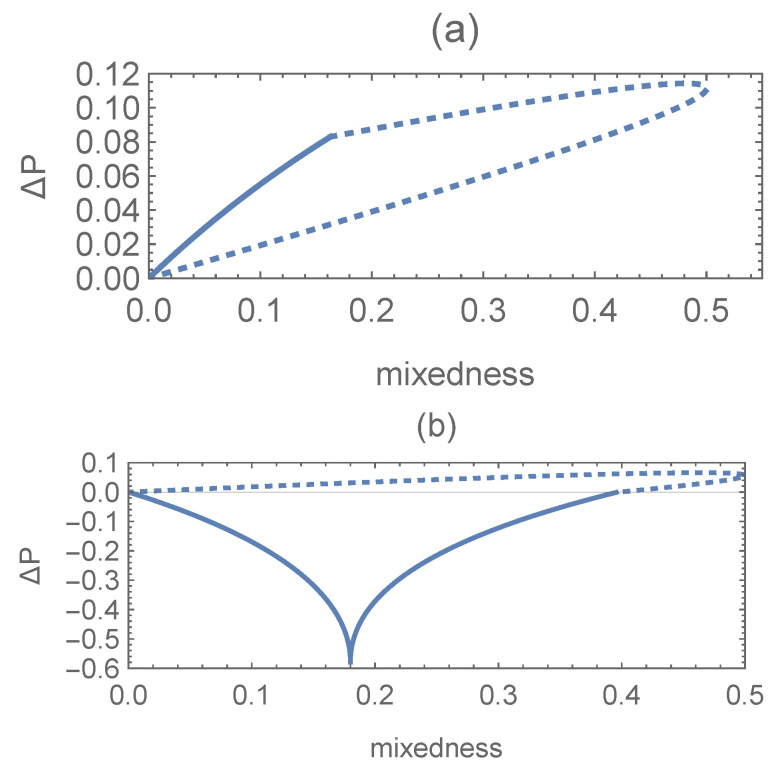
The difference in the optimal success probability ΔP as a function of the linear entropy $S(\rho_{i})$ of the initial states corresponding to the cases of $P_{1}=P_{2}=0.5$ , s=0.1 , and ${\tilde{s}}_{0}=0.9$ . Solid line: $|s^{*}|<3-2\sqrt{2}$ ; dotted line: $|s^{*}|\geq 3-2\sqrt{2}$ . (**a**) $\phi_{1}=\phi_{2}=0$ ; (**b**) $\phi_{1}=\pi /2$ and $\phi_{2}=-\pi /2$ .

The difference between the optimal success probabilities of SD for pure–pure and mixed–mixed states against the linear entropy $S\left(\rho \right)=1-\mathrm{Tr}\rho^{2}$ (mixability) of the initial state is displayed in Figure 3. For $\phi_{1}=\phi_{2}=0$ , the case of an equal-fidelity relation (19) is shown in Figure 3a. We find that the difference ΔP increases with the mixability of the initial mixed states, which is more evident for $s^{*}<3-2\sqrt{2}$ .

If $\phi_{2}\neq \phi_{1}+2k\pi$ for integer *k*, according to Equation (18), one has

(25) $$\begin{array}{c} F(|\Psi_{1}\rangle ,|\Psi_{2}\rangle )<F\left(\rho_{1},\rho_{2}\right). \end{array}$$

Set $\phi_{1}=\pi /2$ and $\phi_{2}=-\pi /2$ . This unequal-fidelity case is shown in Figure 3b. We find that ΔP<0 for r<0.4 . That is, the conclusion in *Theorem 1* no longer holds for these unequal-fidelity cases, which is completely different from the results (Theorem 1) in [23]. Interestingly, for this case of state discrimination with only one observer [23], as the equal-fidelity condition is not satisfied, the relation ΔP≥0 (for the equal-fidelity case) turns into ΔP>0 (for the unequal-fidelity case). Namely, in the framework of SD, the numerical relationship of the results for pure and mixed states is more reliable under the equal-fidelity condition than under the one for the single-time state discrimination [23].

**Figure 4:**
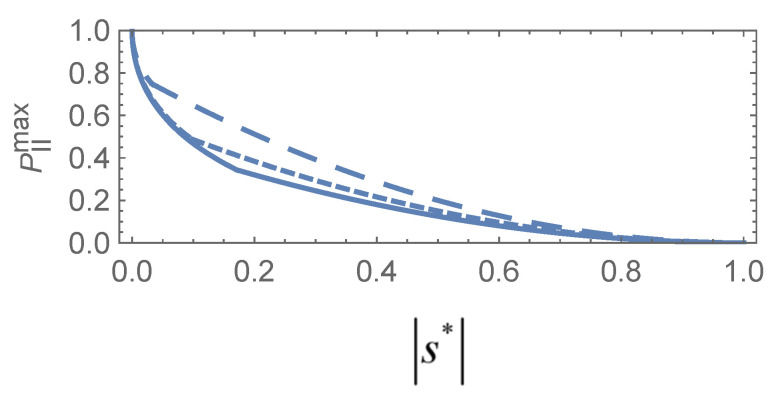
The joint optimal success probability $P_{\mathrm{II}}^{\max}$ as a function of parameter $|s^{*}|$ for $P_{1}=0.5$ (solid line), 0.4 (dotted line), 0.2 (dashed line).

The SD problem with a pure initial state for arbitrary prior probabilities was discussed in a previous work [17], as shown in Figure 4. Based on these results, we can generalize the conclusion of Lemma 1 for equal prior cases to the following proposition.

**Proposition** **1.** For all $q_{0}^{*}\in \left(0,1\right)$ , we have $P_{1}{\left(1-q_{0}^{*}\right)}^{2}+P_{2}{\left(1-\frac{|s^{*}|}{q_{0}^{*}}\right)}^{2}\geq P_{2}\left(1-\right|s^{*}{\left|\right)}^{2}$ if $0<|s^{*}|\leq s_{0}^{*}$ , and $P_{1}{\left(1-q_{0}^{*}\right)}^{2}+P_{2}{\left(1-\frac{|s^{*}|}{q_{0}^{*}}\right)}^{2}<P_{2}\left(1-\right|s^{*}{\left|\right)}^{2}$ if $s_{0}^{*}<\left|s^{*}\right|<1$ .

**Figure 5:**
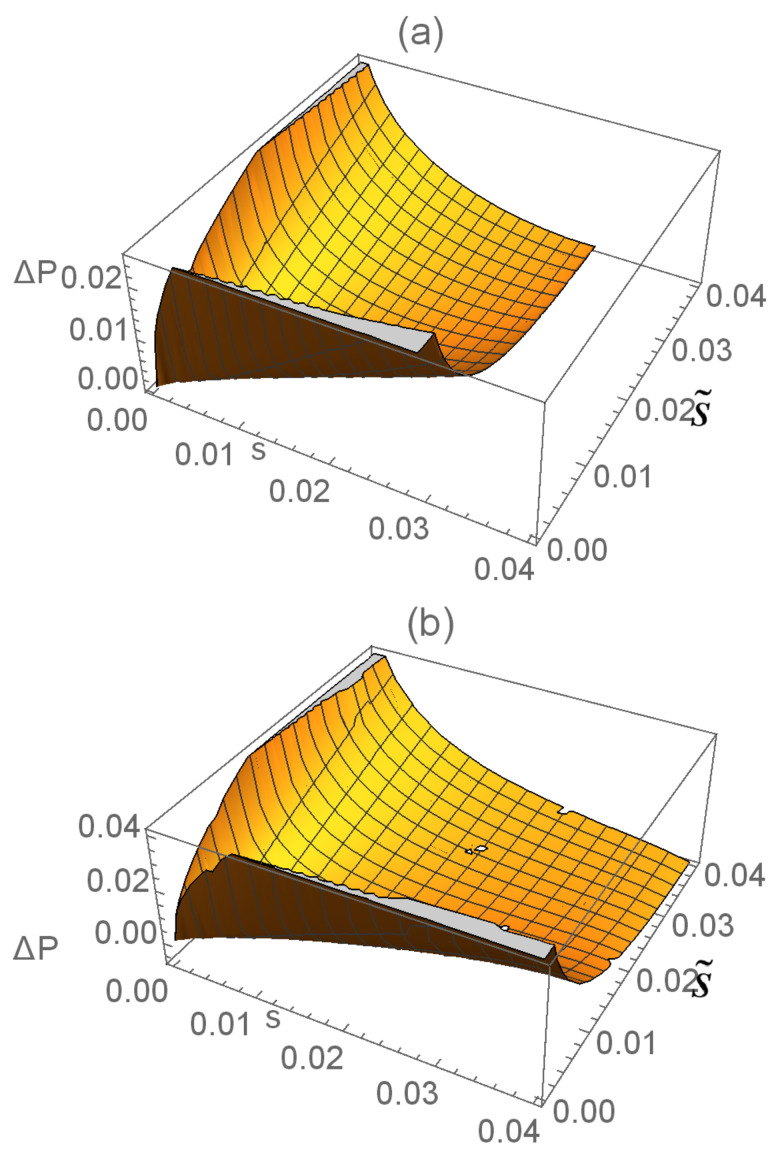
The difference in the optimal success probability ΔP as a function of the overlaps *s* and $\tilde{s}$ corresponding to the unequal-prior cases: (**a**) $P_{1}=1/5$ , $r_{1}=1/5$ , $r_{2}=3/10$ ; (**b**) $P_{1}=r_{1}=r_{2}=1/4$ .

Next, we consider the comparison of the SD for pure and mixed states with arbitrary prior probabilities. Set $S=(s,\tilde{s})$ . Generally, from the complicated expressions in Equations (9) and (15), it is challenging to analytically derive the relationship between $P_{\max}^{I}$ and $P_{\max}^{\mathrm{II}}$ for S∈Λ , where $\Lambda =\{S:0<s\leq s_{0},0<\tilde{s}\leq {\tilde{s}}_{0}\}$ . By performing a numerical experiment involving $10^{5}$ random numbers, we find that the difference $\Delta P=P_{\max}^{I}-P_{\max}^{\mathrm{II}}$ is still non-negative for S∈Λ ; see ΔP as a function of the overlaps *s* and $\tilde{s}$ corresponding to these unequal-prior cases in Figure 5.

For the rest of the cases ( S∉Λ ), based on Equations (9) and (15), we can prove that $\Delta P=P_{\max}^{I}-P_{\max}^{\mathrm{II}}\geq 0$ along with the conclusion presented in Proposition 1. The proof is analogous to that used in Theorem 1 for cases (ii), (iii), and (iv).

## 4. Comparison with Other Scenarios

The sequential discrimination protocol [13] refers to the sequential process for discriminating between quantum states for multiple receivers. This protocol is particularly relevant to scenarios where quantum states cannot be perfectly copied or cloned due to the no-cloning theorem of quantum mechanics. Once classical communication between the receivers is allowed, the quantum nature of the information may be weakened. In this section, we compare the pure and mixed states’ discrimination in two additional scenarios with classical communications [21,23]. The goal here is to determine whether classical mixtures still provide an advantage in these non-SD schemes.

(1) Reproducing protocol: Bob performs an optimal unambiguous discrimination measurement on the quantum state $\rho_{i}$ , which has the same expression as in Equation (1). If he succeeds, he reproduces the state and shares it with Charlie; if he fails, he informs Charlie, and the procedure ends. For this protocol, both Bob and Charlie can perform optimal measurement. Hence, the probability of Bob and Charlie discriminating $\rho_{1}$ and $\rho_{2}$ is PIRe=(1−rs−r˜s˜)2 [21].

(2) Discrimination after broadcasting: Broadcasting [21,26], which is equivalent to the quantum cloning [27] for pure initial states, transforms a mixed state ρ into $\rho_{BC}$ such that ${\mathrm{Tr}}_{B}\rho_{BC}={\mathrm{Tr}}_{C}\rho_{BC}=\rho$ with a certain success probability. If Bob succeeds in broadcasting, he shares state $\rho_{BC}$ with Charlie, and they both perform optimal unambiguous discrimination on their states. Otherwise, if the broadcasting fails, Bob informs Charlie, and the procedure ends. By comparing the discriminations of the pure state with the mixed state with equal priors for the above-mentioned two protocols, we have the following theorem.

**Theorem** **2.** *For the reproducing (discrimination after broadcasting) scheme, the success probability PIIRe ( $P_{\mathrm{II}}^{\mathrm{Br}}$ ) of optimally discriminating the mixed states $\left\{\rho_{1},\rho_{2}\right\}$ given in (1) is less than or equal to PIRe ( $P_{I}^{\mathrm{Br}}$ ) for the pure states $\{|\Psi_{1}\rangle ,|\Psi_{2}\rangle \}$ given in (11).*

**Proof** **of** **Theorem** **2.** For the reproducing scenario, according to [13,21], we have

(26) PIRe=(1−rs−r˜s˜)2≤{1−|rs+exp[i(ϕ2−ϕ1)]r˜s˜|}2=(1−|s*|)2=PIIRe,

As $\phi_{1}=\phi_{2}$ (the equal-fidelity condition is satisfied), we have PIRe=PIIRe.

For discrimination after broadcasting, according to the results in [13,21], one has

(27) $$P_{I}^{\mathrm{Br}}=\min\left\{\frac{1}{1+s},\frac{1}{1+\tilde{s}}\right\}{\left(1-rs-\tilde{r}\tilde{s}\right)}^{2},$$

which was calculated in Theorem 2 in [21].

Since $\min\left\{\frac{1}{1+s},\frac{1}{1+\tilde{s}}\right\}\leq \frac{1}{1+rs+\tilde{r}\tilde{s}},$ from (18), (27), and the monotonically decreasing properties of the function $f\left(x\right)=\frac{{\left(1-x\right)}^{2}}{1+x}$ for x∈[0,1] , we obtain

(28) $$\begin{array}{ccc} P_{I}^{\mathrm{Br}} & \leq & \frac{{\left(1-rs-\tilde{r}\tilde{s}\right)}^{2}}{1+rs+\tilde{r}\tilde{s}}\\ & \leq & \frac{\left(1-\right|s^{*}{\left|\right)}^{2}}{1+|s^{*}|}=P_{\mathrm{II}}^{\mathrm{Br}}. \end{array}$$

As $\phi_{1}=\phi_{2}$ and $s=\tilde{s}$ (the equal-fidelity condition is satisfied), we have PIRe=PIIRe. □

In the framework of these two scenarios with classical communications, all of the measurements involved are optimal, which is completely different from the one for SD. Since all of the information is extracted after the first measurement, different from the SD scheme, the advantages of mixed state discrimination disappear here. In order to clarify the difference between pure–pure and mixed–mixed schemes under different physical backgrounds in the framework of SD, we consider the classical mutual information in the following section.

## 5. Mutual Information

Classical mutual information quantifies the information transmitted between the involved parties [18]. Let *X* (*Y*) denote the message of the sender *A* (receiver *B*). The mutual information of the communication channel is defined as

I(A:B)=H(X)−H(X|Y),

where H(X) is the Shannon entropy,

H(X)=−XlogX−(1−X)log(1−X),

and H(X|Y) is the conditional Shannon entropy.

In the framework of SD, the mutual information between Bob and Charlie takes into account the events where both of them have successfully identified Alice’s message so that they share an identical string of qudits. Hence, the mutual information between Bob and Charlie for the unambiguous communication channel can be written as [18],

(29) $$I\left(B:C\right)=P_{I}\left[H\left(X_{c}\right)-H\left(X_{c}|Y_{c}\right)\right],$$

where $X_{c}$ and $Y_{c}$ denote the information carried by Bob and Charlie. Hence, after discarding the failure results, the outcome is conclusive with the probability $P_{I}$ expressed in Equation (6). Then, one has $H(X_{c}|Y_{c})=0$ because there is no uncertainty without the inclusive result [18]. When Charlie attempts to detect states $\left\{\rho_{1},\rho_{2}\right\}$ , the prior probability for Bob’s message $X_{c}$ is given by confidence probabilities $\{C_{s},1-C_{s}\},$ where

$$C_{s}=\frac{P_{1}r_{1}\left(1-q_{1}^{b}\right)\left(1-q_{1}^{c}\right)+P_{1}{\tilde{r}}_{1}\left(1-{\tilde{q}}_{1}^{b}\right)\left(1-{\tilde{q}}_{1}^{c}\right)}{P_{I}}.$$

Then, the mutual information between Bob and Charlie for this unambiguous communication channel (30) is given by

(30) $$I_{I}\left(B:C\right)=P_{I}H\left(C_{s}\right).$$

The mutual information is proven to be a quantity consistent with the unambiguous discrimination scheme. Its upper bound coincides with the optimal successful probability of SD for pure states [18]. After the above extension to the SD of mixed states, we consider the special properties of the mixed states in Equation (1), where the two groups of vectors lying in their respective subspaces are orthogonal to each other, and the successful probability of the SD for the mixed states is equivalent to a weighted value of the one for the basic vectors $|R_{1}\rangle$ , $|R_{2}\rangle$ , and $|{\tilde{R}}_{1}\rangle$ , $|{\tilde{R}}_{2}\rangle$ [21,23]. Namely, we have

(31) $$P_{I}=\left(P_{1}r_{1}+P_{2}r_{2}\right)P_{\mathrm{II}}^{\left(1,2\right)}+\left(P_{1}{\tilde{r}}_{1}+P_{2}{\tilde{r}}_{2}\right)P_{\mathrm{II}}^{\left(\tilde{1},\tilde{2}\right)},$$

where $P_{\mathrm{II}}^{\left(1,2\right)}$ and $P_{\mathrm{II}}^{\left(\tilde{1},\tilde{2}\right)}$ demonstrate the successful probability of the SD of the pure states { $|R_{1}\rangle$ , $|R_{2}\rangle$ } and { $|{\tilde{R}}_{1}\rangle$ , $|{\tilde{R}}_{2}\rangle$ }, prepared with prior probabilities $P_{1}^{0},P_{2}^{0}$ and ${\tilde{P}}_{1}^{0},{\tilde{P}}_{2}^{0}$ , respectively, with

$$P_{i}^{0}=\frac{P_{i}r_{i}}{P_{1}r_{1}+P_{2}r_{2}},\;{\tilde{P}}_{i}^{0}=\frac{P_{i}{\tilde{r}}_{i}}{P_{1}{\tilde{r}}_{1}+P_{2}{\tilde{r}}_{2}},\;i=1,2.$$

Concerning the relations of the mutual information occurring in the SD for both pure and mixed states, we have the following theorem.

**Theorem** **3.** *Mutual information $I_{I}$ for the SD of mixed states in (1) between the subsequent observers Bob and Charlie satisfies the following relation:*

$$I_{I}\geq \left(P_{1}r_{1}+P_{2}r_{2}\right)I_{\mathrm{II}}^{\left(1,2\right)}+\left(P_{1}{\tilde{r}}_{1}+P_{2}{\tilde{r}}_{2}\right)I_{\mathrm{II}}^{\left(\tilde{1},\tilde{2}\right)},$$

*where $I_{\mathrm{II}}^{\left(1,2\right)}$ and $I_{\mathrm{II}}^{\left(\tilde{1},\tilde{2}\right)}$ demonstrate the mutual information between the two successive observers for the SD of the pure states { $|R_{1}\rangle$ , $|R_{2}\rangle$ } and { $|{\tilde{R}}_{1}\rangle$ , $|{\tilde{R}}_{2}\rangle$ }, respectively.*

**Proof** **of** **Theorem** **3.** Set

$$F_{i}=P_{i}r_{i}\left(1-q_{i}^{b}\right)\left(1-q_{i}^{c}\right),\;\;{\tilde{F}}_{i}=P_{i}{\tilde{r}}_{i}\left(1-{\tilde{q}}_{i}^{b}\right)\left(1-{\tilde{q}}_{i}^{c}\right).$$

We have

(32) $$P_{I}=\sum_{i=1}^{2}F_{i}+{\tilde{F}}_{i}.$$

According to (30) and (32), the mutual information between Bob and Charlie can be expressed as

(33) $$\begin{array}{ccc} I_{I} & \;=\; & -P_{I}[\frac{F_{1}+{\tilde{F}}_{1}}{\sum_{i=1}^{2}F_{i}+{\tilde{F}}_{i}}\log_{2}\left(\frac{F_{1}+{\tilde{F}}_{1}}{\sum_{i=1}^{2}F_{i}+{\tilde{F}}_{i}}\right)\\ & & +\frac{F_{2}+{\tilde{F}}_{2}}{\sum_{i=1}^{2}F_{i}+{\tilde{F}}_{i}}\log_{2}\left(\frac{F_{2}+{\tilde{F}}_{2}}{\sum_{i=1}^{2}F_{i}+{\tilde{F}}_{i}}\right)]\\ & \;=\; & P_{I}\{-\frac{F_{1}\;+\;{\tilde{F}}_{1}}{\sum_{i=1}^{2}F_{i}\;+\;{\tilde{F}}_{i}}\log_{2}\left[P_{x}\frac{F_{1}}{F_{1}\;+\;F_{2}}+\left(1\;-\;P_{x}\right)\frac{{\tilde{F}}_{1}}{{\tilde{F}}_{1}\;+\;{\tilde{F}}_{2}}\right]\\ & & -\frac{F_{2}\;+\;{\tilde{F}}_{2}}{\sum_{i=1}^{2}F_{i}\;+\;{\tilde{F}}_{i}}\log_{2}\left[P_{x}\frac{F_{2}}{F_{1}\;+\;F_{2}}+\left(1\;-\;P_{x}\right)\frac{{\tilde{F}}_{2}}{{\tilde{F}}_{1}\;+\;{\tilde{F}}_{2}}\right]\}\\ & \;=\; & P_{I}H\left[P_{x}\frac{F_{1}}{F_{1}\;+\;F_{2}}\;+\;\left(1\;-\;P_{x}\right)\frac{{\tilde{F}}_{1}}{{\tilde{F}}_{1}\;+\;{\tilde{F}}_{2}}\right], \end{array}$$

with $P_{x}=\frac{F_{1}+F_{2}}{F_{1}+{\tilde{F}}_{1}+F_{2}+{\tilde{F}}_{2}}.$ The expressions of $I_{I}^{\left(1,2\right)}$ and $I_{I}^{\left(\tilde{1},\tilde{2}\right)}$ are given by

$$I_{\mathrm{II}}^{\left(1,2\right)}=\sum_{i=1}^{2}P_{i}^{0}\left(1-q_{i}^{b}\right)\left(1-q_{i}^{c}\right)H\left(\frac{F_{1}}{F_{1}+F_{2}}\right),$$

$$I_{\mathrm{II}}^{\left(\tilde{1},\tilde{2}\right)}=\sum_{i=1}^{2}P_{i}^{0}\left(1-{\tilde{q}}_{i}^{b}\right)\left(1-{\tilde{q}}_{i}^{c}\right)H\left(\frac{{\tilde{F}}_{1}}{{\tilde{F}}_{1}+{\tilde{F}}_{2}}\right).$$

According to Equation (33) and the concavity of entropy function H(x) , one has

(34) $$\begin{array}{ccc} I_{I} & \geq & P_{I}\left[P_{x}H\left(\frac{F_{1}}{F_{1}\;+\;F_{2}}\right)+\left(1-P_{x}\right)H\left(\frac{{\tilde{F}}_{1}}{{\tilde{F}}_{1}\;+\;{\tilde{F}}_{2}}\right)\right]\\ & = & \left(P_{1}r_{1}+P_{2}r_{2}\right)I_{\mathrm{II}}^{\left(1,2\right)}+\left(P_{1}{\tilde{r}}_{1}+P_{2}{\tilde{r}}_{2}\right)I_{\mathrm{II}}^{\left(\tilde{1},\tilde{2}\right)}. \end{array}$$

The equality holds for either $P_{x}=0$ or 1. □

Since the upper bounds of mutual information equal those of the optimal success probabilities of the SD for the pure states [18], we have

(35) $$\sup I_{\mathrm{II}}^{\left(1,2\right)}=P_{\mathrm{II}}^{\left(1,2\right)},\;\;\;\sup I_{\mathrm{II}}^{\left(\tilde{1},\tilde{2}\right)}=P_{\mathrm{II}}^{\left(\tilde{1},\tilde{2}\right)}.$$

For the SD of pure superposed states given in the form in Equation (11), according to the relations (30), (34), and (35), we have the following corollary.

**Corollary** **1.** *The optimal mutual information between Bob and Charlie is larger than the maximal success probability of SD for mixed states, namely, $\sup I_{I}\geq P_{I}$ .*

**Figure 6:**
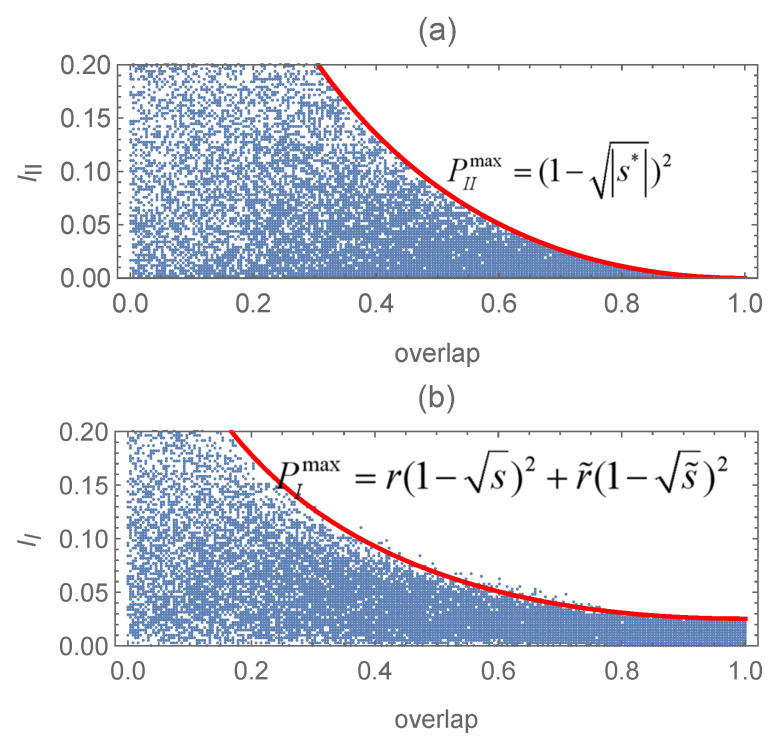
Mutual information between Bob and Charlie against the overlap ( $|s^{*}|$ in (**a**) and *s* in (**b**)) corresponding to the equal-prior case ( $P_{1}=P_{2}=1/2$ ). (**a**) Pure–pure scheme; (**b**) mixed–mixed scheme with $r_{1}=r_{2}=1/2$ , $\tilde{s}=0.6$ . The red line corresponds to the optimal success probability of state discrimination.

We illustrate the corollary in Figure 6. For the SD of the pure state with equal prior, the result is inconsistent with that in [18], as shown in Figure 6a. The mutual information between Bob and Charlie is upper-bounded by the optimal success probability of SD, $P_{\mathrm{II}}^{\max}={\left(1-\sqrt{|s^{*}|}\right)}^{2}$ , which is saturated at $q_{1}^{b}=q_{1}^{c}=\sqrt{|s^{*}|}.$ For the SD of the mixed states shown in Figure 6b, it is indicated that the optimal success probability of SD ( $P_{I}^{\max}=r{\left(1-\sqrt{s}\right)}^{2}+\tilde{r}{\left(1-\sqrt{\tilde{s}}\right)}^{2}$ shown in Table I) is no longer a compact upper bound but an approximate estimation of the optimal mutual information between Bob and Charlie. The optimal success probability of SD is less than the mutual information. Generally, we conclude that classical mixture of basic vectors due to quantum decoherence has a subtle influence on the mutual information in SD schemes.

## 6. Conclusions

We investigated the SD of two mixed states and compared the optimal joint success probability of the two observers with that of the SD for two pure states superposed via the basic vectors of the mixed states. Since there is more residual information left after the first measurement in the SD procedure of mixed states than in the case of pure states, different from the existing results in [23,24], we showed that the result of coherent superposition is inferior to that of classical mixing contrarily. This result is seriously dependent on equal-fidelity conditions. In addition, since there are essential differences between classical and quantum channels, we considered another two scenarios with classical communications. Different from the results from SD schemes, it was shown that the pure–pure scheme is superior to the mixed–mixed one, because the measurements performed by each observer are simply optimal.

In order to clarify the difference between pure–pure and mixed–mixed state discrimination schemes in the framework of SD, we studied the classical mutual information between two successive observers. Since the mutual information for the SD of pure states was calculated in [28], where the maximum of the mutual information conditioned on success was found to coincide with the maximum of the success of probability of SD [18], the sequential unambiguous discrimination strategy can be employed as a multiparty communication channel [18]. Then, for the SD of mixed states, we derived a theorem that indicates that the mutual information for the SD of mixed states is superior to the weighted value of that for the SD of the two basic vectors. Hence, we inferred that the optimal mutual information between Bob and Charlie is superior to the maximal success probability of SD for mixed states. We concluded that the classical mixture of basic vectors from quantum decoherence has a subtle effect on the communication channel, which was included in an SD scheme for pure states and expressed as the coincidence of maximal mutual information and optimal successful probability.

The comparison between pure state and mixed state SD is highly significant in the context of quantum state discrimination. In practical quantum communication and computation, mixed states often arise due to decoherence. Our findings suggest that the advantages of state discriminations can be present in mixed state discrimination protocols, which may help with developing more effective strategies for tasks like quantum key distribution where distinguishing between different quantum states is crucial. Our approaches also highlight related research on, for example entanglement and coherence, with similar findings to those given in [23,24].

However, there is one limitation of our work that warrants future improvement. The assumption that the support spaces of the two states do not overlap simplifies the discrimination process, but it may not be applicable in all practical scenarios. Future research could focus on methods that handle overlapping support spaces by developing more advanced algorithms. The generalization may be of considerable significance.

## Figures and Tables

**Table 1:** Optimal success probability $P_{I}^{\max}$ of SD for symmetric mixed state with equal prior in terms of *r*, $\tilde{r}$ , *s*, and $\tilde{s}$ . The results correspond to four cases for the value of *s* and $\tilde{s}$ and are the same as those in [21].

Overlap	$s>3-2\sqrt{2}$	$s\leq 3-2\sqrt{2}$
$\tilde{s}>3-2\sqrt{2}$	$P_{I}^{\max}=\frac{1}{2}r{\left(1-s\right)}^{2}+\frac{1}{2}\tilde{r}{\left(1-\tilde{s}\right)}^{2}$	$P_{I}^{\max}=r{\left(1-\sqrt{s}\right)}^{2}+\frac{1}{2}\tilde{r}{\left(1-\tilde{s}\right)}^{2}$
$\tilde{s}\leq 3-2\sqrt{2}$	$P_{I}^{\max}=\frac{1}{2}r{\left(1-s\right)}^{2}+\tilde{r}{\left(1-\sqrt{\tilde{s}}\right)}^{2}$	$P_{I}^{\max}=r{\left(1-\sqrt{s}\right)}^{2}+\tilde{r}{\left(1-\sqrt{\tilde{s}}\right)}^{2}$

## Data Availability

No new data were created or analyzed in this study. Data sharing is not applicable to this article.

## Appendix A. POVM for SD of Mixed States

We now consider the procedure of mixed state discrimination via the language of POVM. Alice prepares the mixed state (1) and sends it to Bob. Bob performs an operation with POVM operators

(A1)
$$\begin{array}{cc} M_{1}^{b} & =c_{1}|R_{2}^{⊥}\rangle \langle R_{2}^{⊥}|+{\tilde{c}}_{1}|{\tilde{R}}_{2}^{⊥}\rangle \langle {\tilde{R}}_{2}^{⊥}|,\\ M_{2}^{b} & =c_{2}|R_{1}^{⊥}\rangle \langle R_{1}^{⊥}|+{\tilde{c}}_{2}|{\tilde{R}}_{1}^{⊥}\rangle \langle {\tilde{R}}_{1}^{⊥}|,\\ M_{0}^{b} & =I^{b}-M_{1}^{b}-M_{2}^{b}, \end{array}$$

where { $|R_{1}^{⊥}\rangle ,\;|{\tilde{R}}_{1}^{⊥}\rangle$ } and { $|R_{2}^{⊥}\rangle ,\;|{\tilde{R}}_{2}^{⊥}\rangle$ } are bases orthogonal to { $|R_{1}\rangle ,\;|{\tilde{R}}_{1}\rangle$ } and { $|R_{2}\rangle ,\;|{\tilde{R}}_{2}\rangle$ }, respectively. $c_{i}$ and ${\tilde{c}}_{i}$ are non-negative real numbers less than 1. Bob’s POVM (A1) must satisfy the following properties: $M_{i}^{b}\geq 0$ ( i=0,1,2 ), $M_{1}^{b}+M_{2}^{b}+M_{0}^{b}=I^{b}$ and $\mathrm{Tr}[\rho_{i}\left(M_{j}^{B}\right)]=0$ for i≠j=1,2 , in which the first two relations represent the positive semidefiniteness and completeness conditions of the POVM, respectively; the last relation ensures that there is no error in state discrimination.

The operators $M_{1}^{b}$ and $M_{2}^{b}$ correspond to the conclusive outcomes (indicating successful results), whereas $M_{0}^{b}$ represents the inconclusive outcome (indicating failure). From assumptions (2) and (3), the subspace spanned by { $|R_{1}^{⊥}\rangle ,\;|R_{2}^{⊥}\rangle$ } is orthogonal to the one spanned by $\{|{\tilde{R}}_{1}^{⊥}\rangle ,\;|{\tilde{R}}_{2}^{⊥}\rangle \}$ . Thus, the POVM in (A1) can be written as

(A2)
$$\begin{array}{ccc} M_{1}^{b} & = & c_{1}|R_{2}^{⊥}\rangle \langle R_{2}^{⊥}|⊕{\tilde{c}}_{1}|{\tilde{R}}_{2}^{⊥}\rangle \langle {\tilde{R}}_{2}^{⊥}|,\\ M_{2}^{b} & = & c_{2}|R_{1}^{⊥}\rangle \langle R_{1}^{⊥}|⊕{\tilde{c}}_{2}|{\tilde{R}}_{1}^{⊥}\rangle \langle {\tilde{R}}_{1}^{⊥}|,\\ M_{0}^{b} & = & M_{0s}^{b}⊕{\tilde{M}}_{0s}^{b}\\ & = & (I_{s}^{b}-c_{1}|R_{1}^{⊥}\rangle \langle R_{1}^{⊥}|-c_{2}|R_{2}^{⊥}\rangle \langle R_{2}^{⊥}|)⊕({\tilde{I}}_{s}^{b}-{\tilde{c}}_{1}|{\tilde{R}}_{1}^{⊥}\rangle \langle {\tilde{R}}_{1}^{⊥}|-{\tilde{c}}_{2}|{\tilde{R}}_{2}^{⊥}\rangle \langle {\tilde{R}}_{2}^{⊥}|). \end{array}$$

Here, $I_{s}^{b}$ and ${\tilde{I}}_{s}^{b}$ denote the identity matrices on their respective subspaces. The POVM (A2) ensures that the discrimination of mixed states can be performed in two separate and independent subspaces.

Set $q_{i}^{b}=\langle R_{i}|M_{0s}^{b}|R_{i}\rangle$ and ${\tilde{q}}_{i}^{b}=\langle {\tilde{R}}_{i}|{\tilde{M}}_{0s}^{b}|{\tilde{R}}_{i}\rangle$ . We have

(A3)
$$q_{i}^{b}=1-c_{i}\left(1-s^{2}\right),\;\;\;{\tilde{q}}_{i}^{b}=1-{\tilde{c}}_{i}\left(1-{\tilde{s}}^{2}\right),$$

where $s^{2}\leq q_{i}^{b}\leq 1$ , and ${\tilde{s}}^{2}\leq {\tilde{q}}_{i}^{b}\leq 1$ ( i=1,2 ).

As a positive semidefinite operator, the POVM operator $M_{0}^{b}$ satisfies $\det M_{0}^{b}\geq 0$ [21], which requires

(A4)
$$\begin{array}{c} q_{1}^{b}q_{2}^{b}-s^{2}\geq 0,\;\;\;{\tilde{q}}_{1}^{b}{\tilde{q}}_{2}^{b}-{\tilde{s}}^{2}\geq 0. \end{array}$$

If Bob extracts all the information encoded in the first particle of the bipartite state, the relations (A4) hold as equalities. Conversely, if he does not extract all the information, they become strict inequalities.

The Kraus operators $K_{i}^{b}$ ( i=0,1,2 ) corresponding to Bob’s POVM ( $M_{i}^{b}=K_{i}^{b†}K_{i}^{b}$ ) are given by [21]

(A5)
$$\begin{array}{ccc} \; & K_{1}^{b}=\sqrt{c_{1}}|V_{1}\rangle \langle R_{2}^{⊥}|+\sqrt{{\tilde{c}}_{1}}|{\tilde{V}}_{1}\rangle \langle {\tilde{R}}_{2}^{⊥}|, & \\ \; & K_{2}^{b}=\sqrt{c_{2}}|V_{2}\rangle \langle R_{1}^{⊥}|+\sqrt{{\tilde{c}}_{2}}|{\tilde{V}}_{2}\rangle \langle {\tilde{R}}_{1}^{⊥}|, & \\ \; & K_{0}^{b}=(\sqrt{a_{1}}|V_{1}\rangle \langle R_{2}^{⊥}|+\sqrt{a_{2}}|V_{2}\rangle \langle R_{1}^{⊥}|)⊕(\sqrt{{\tilde{a}}_{1}}|{\tilde{V}}_{1}\rangle \langle {\tilde{R}}_{2}^{⊥}|+\sqrt{{\tilde{a}}_{2}}|{\tilde{V}}_{2}\rangle \langle {\tilde{R}}_{1}^{⊥}|), \end{array}$$

where $a_{i}=q_{i}^{b}/\left(1-s^{2}\right)$ , ${\tilde{a}}_{i}={\tilde{q}}_{i}^{b}/\left(1-{\tilde{s}}^{2}\right)$ , and i=1,2 .

The post-measured state $\sigma_{i}$ ( i=1,2 ), which corresponds to Bob’s success result, can be expressed as

(A6)
$$\begin{array}{c} \sigma_{i}=\frac{K_{i}^{b}\rho_{i}K_{i}^{b†}}{\mathrm{Tr}(K_{i}^{b}\rho_{i}K_{i}^{b†})}=v_{i}|V_{i}\rangle \langle V_{i}|+{\tilde{v}}_{i}|{\tilde{V}}_{i}\rangle \langle {\tilde{V}}_{i}|, \end{array}$$

where { $|V_{i}\rangle ,\;|{\tilde{V}}_{i}\rangle$ } is the orthonormal basis of $\sigma_{i}$ satisfying

(A7)
$$\begin{array}{cc} \; & \langle V_{1}|V_{2}\rangle =t,\;\langle {\tilde{V}}_{1}|{\tilde{V}}_{2}\rangle =\tilde{t},\;\langle V_{1}|{\tilde{V}}_{2}\rangle =\langle {\tilde{V}}_{1}|V_{2}\rangle =0. \end{array}$$

The eigenvalues of $\sigma_{i}$ are

(A8)
$$\begin{array}{c} v_{i}=\frac{\left(1-q_{i}^{b}\right)r_{i}}{\left(1-q_{i}^{b}\right)r_{i}+\left(1-{\tilde{q}}_{i}^{b}\right){\tilde{r}}_{i}},\;\;\;{\tilde{v}}_{i}=\frac{\left(1-{\tilde{q}}_{i}^{b}\right){\tilde{r}}_{i}}{\left(1-q_{i}^{b}\right)r_{i}+\left(1-{\tilde{q}}_{i}^{b}\right){\tilde{r}}_{i}}, \end{array}$$

respectively.

The equivalence of the two expressions (A2) and (A5) ( $M_{0}^{A}=K_{0}^{A†}K_{0}^{A}$ ) requires

(A9)
$$q_{1}^{b}q_{2}^{b}=s^{2}/t^{2},\;\;{\tilde{q}}_{1}^{b}{\tilde{q}}_{2}^{b}={\tilde{s}}^{2}/{\tilde{t}}^{2}.$$

One has that $q_{i}^{b}$ ( ${\tilde{q}}_{i}^{b}$ ) is lower-bounded by $s^{2}/t^{2}$ ( ${\tilde{s}}^{2}/{\tilde{t}}^{2}$ ). The success probability of Bob identifying his state is

(A10)
$$\begin{array}{ccc} P^{B} & = & \sum_{i=1}^{2}P_{i}\mathrm{Tr}\left(\rho_{i}M_{i}^{b}\right)\\ & = & \sum_{i=1}^{2}\left[P_{i}r_{i}\left(1-q_{i}^{b}\right)+P_{i}{\tilde{r}}_{i}\left(1-{\tilde{q}}_{i}^{b}\right)\right]. \end{array}$$

Based on Equations (A4) and (A9), the optimal discrimination is achieved when $t=\tilde{t}=1$ . Consequently, the relation (A9) becomes

(A11)
$$q_{1}^{b}q_{2}^{b}=s^{2},\;\;{\tilde{q}}_{1}^{b}{\tilde{q}}_{2}^{b}={\tilde{s}}^{2}$$

with parameters $q_{i}^{b}$ and ${\tilde{q}}_{i}^{b}$ satisfying

(A12)
$$q_{i}^{b}\in \left[s^{2},1\right],\;\;{\tilde{q}}_{i}^{b}\in \left[{\tilde{s}}^{2},1\right].$$

Here, Bob’s POVMs are nonoptimal, indicated by t≠1 and $\tilde{t}\neq 1$ . This means that after Bob’s discrimination, some information still remains in the state. The post-measured state is then sent to another observer, Charlie, who performs discrimination on the same particle using POVMs. The specific POVMs used by Charlie are given by

(A13)
$$\begin{array}{ccc} M_{1}^{c} & = & \frac{1-q_{1}^{c}}{1-t^{2}}|V_{2}^{⊥}\rangle \langle V_{2}^{⊥}|+\frac{1-{\tilde{q}}_{1}^{c}}{1-{\tilde{t}}^{2}}|{\tilde{V}}_{2}^{⊥}\rangle \langle {\tilde{V}}_{2}^{⊥}|,\\ M_{2}^{c} & = & \frac{1-q_{2}^{c}}{1-t^{2}}|V_{1}^{⊥}\rangle \langle V_{1}^{⊥}|+\frac{1-{\tilde{q}}_{2}^{c}}{1-{\tilde{t}}^{2}}|{\tilde{V}}_{1}^{⊥}\rangle \langle {\tilde{V}}_{1}^{⊥}|,\\ M_{0}^{c} & = & I-M_{1}^{c}-M_{2}^{c}, \end{array}$$

where { $|V_{1}^{⊥}\rangle$ , $|{\tilde{V}}_{1}^{⊥}\rangle$ } and { $|V_{2}^{⊥}\rangle$ , $|{\tilde{V}}_{2}^{⊥}\rangle$ } are bases orthogonal to { $|V_{1}\rangle$ , $|{\tilde{V}}_{1}\rangle$ } and { $|V_{2}\rangle$ , $|{\tilde{V}}_{2}\rangle$ }, respectively; $q_{i}^{c}=\langle V_{i}|\Pi_{0}|V_{i}\rangle$ , and ${\tilde{q}}_{i}^{c}=\langle {\tilde{V}}_{i}|\Pi_{0}|{\tilde{V}}_{i}\rangle$ for i=1,2 .

Charlie’s discrimination is optimal in the sense that $\det M_{0}^{c}=0$ , i.e., $q_{1}^{c}q_{2}^{c}-t^{2}=0$ , and ${\tilde{q}}_{1}^{c}{\tilde{q}}_{2}^{c}-{\tilde{t}}^{2}=0$ . The joint success probability of both Bob and Charlie identifying the states is then given by

(A14)
$$\begin{array}{ccc} \;\;P_{I}\;\; & \;\;=\;\;\;\; & \sum_{i=1}^{2}P_{i}\mathrm{Tr}\left[\rho_{i}M_{i}^{b}\right]\mathrm{Tr}\left[\sigma_{i}M_{i}^{c}\right]\\ & \;\;=\;\;\; & \sum_{i=1}^{2}P_{i}\;\left[r_{i}\;\left(1\;\;-\;\;q_{i}^{b}\right)\;\left(1\;\;-\;\;q_{i}^{c}\right)\;\;+\;\;{\tilde{r}}_{i}\;\left(1\;\;-\;\;{\tilde{q}}_{i}^{b}\right)\;\left(1\;\;-\;\;{\tilde{q}}_{i}^{c}\;\right)\;\right], \end{array}$$

which is given in (6).
